# Systems Biology Reveals S-Nitrosylation-Dependent Regulation of Mitochondrial Functions in Mice with *Shank3* Mutation Associated with Autism Spectrum Disorder

**DOI:** 10.3390/brainsci11060677

**Published:** 2021-05-21

**Authors:** Maryam Kartawy, Igor Khaliulin, Haitham Amal

**Affiliations:** Faculty of Medicine, Institute for Drug Research, School of Pharmacy, The Hebrew University of Jerusalem, Jerusalem 91120, Israel; maryam.kartawy@mail.huji.ac.il (M.K.); igorkh@savion.huji.ac.il (I.K.)

**Keywords:** autism spectrum disorder, nitric oxide, S-nitrosylation, proteomics mitochondria, systems biology

## Abstract

Autism spectrum disorder (ASD) is a neurodevelopmental disorder manifested in repetitive behavior, abnormalities in social interactions, and communication. The pathogenesis of this disorder is not clear, and no effective treatment is currently available. Protein S-nitrosylation (SNO), the nitric oxide (NO)-mediated posttranslational modification, targets key proteins implicated in synaptic and neuronal functions. Previously, we have shown that NO and SNO are involved in the ASD mouse model based on the *Shank3* mutation. The energy supply to the brain mostly relies on oxidative phosphorylation in the mitochondria. Recent studies show that mitochondrial dysfunction and oxidative stress are involved in ASD pathology. In this work, we performed SNO proteomics analysis of cortical tissues of the *Shank3* mouse model of ASD with the focus on mitochondrial proteins and processes. The study was based on the SNOTRAP technology followed by systems biology analysis. This work revealed that 63 mitochondrial proteins were S-nitrosylated and that several mitochondria-related processes, including those associated with oxidative phosphorylation, oxidative stress, and apoptosis, were enriched. This study implies that aberrant SNO signaling induced by the *Shank3* mutation can target a wide range of mitochondria-related proteins and processes that may contribute to the ASD pathology. It is the first study to investigate the role of NO-dependent mitochondrial functions in ASD.

## 1. Introduction

Autism spectrum disorder (ASD) is a neurodevelopmental disorder characterized by early-onset deficits in social communication, repetitive behaviors, and restricted interests [1,2]. The etiology and biological basis of ASD are still unclear. However, many genetic mutations and non-genetic and environmental factors have been linked to ASD [1]. *SHANK3* mutation is one of the most promising ASD-associated human mutations. Previous experiments on *Shank3* knockout mouse models showed defects in cellular, biochemical, and electrophysiological pathways [3,4,5]. In this work, we used the human mutation-based mouse model for ASD (InsG3680(+/+)), in which a guanine nucleotide was inserted at cDNA position 3680 of the *Shank3* gene, leading to a frameshift that resulted in a premature stop codon. These mice were shown to have striatal and cortico-striatal synaptic transmission and social behavioral defects, along with intense overgrooming and repetitive behavior [5].

Over recent years, evidence has emerged indicating that ASD implicates non-neuronal processes, such as mitochondrial dysfunction, oxidative stress, gastrointestinal abnormalities, and abnormal immune system regulation [6,7,8].

Thus, several lines of observations have implicated the mitochondria in the pathophysiology of ASD [6,9,10,11]. Mitochondria are distinct cellular organelles responsible for the oxidative conversion of macronutrients, amino acids, fatty acids, and pyruvate, into adenosine triphosphate (ATP). Mitochondria generate energy via oxidative phosphorylation (OXPHOS). These organelles also regulate calcium homeostasis and signaling, apoptosis, cellular stress responses, the formation of reactive oxygen species (ROS), and redox signaling [12,13].

Since the brain accounts for 20% of the body’s metabolism and developing neurons primarily rely on the OXPHOS for essential neurodevelopmental processes, the immature nervous system is remarkably vulnerable to defects in the bioenergetic capacity [6,14]. Therefore, it is not surprising that mitochondrial dysfunction may be involved in the etiology of ASD.

One of the regulators of neuronal energy production by the mitochondria, as well as many other functions in the brain, is nitric oxide (NO) [15]. NO is produced in the central and peripheral nervous systems [3,16], mainly from L-arginine by an enzymatic activity carried out by three isoforms of nitric oxide synthase, neuronal (nNOS or NOS1), endothelial (eNOS or NOS2), and inducible (iNOS or NOS3) NO synthase [16,17]. At low concentrations, NO serves as a signaling molecule. It plays an essential role in the regulation of synaptic activity, synaptic plasticity, and vesicle trafficking [3,16,17]. However, at high concentrations, NO may exert toxic effects, possibly leading to modified phenotypes and cell death [16]. NO reacts with superoxide radical (O_2−_) forming peroxynitrite (ONOO-), which ultimately induces damage to DNA, lipids, and proteins during oxidative stress. NO affects cellular signaling through proteins S-nitrosylation (SNO), tyrosine nitration, and S-nitrosoglutathione (GSNO) formation. SNO is a reversible NO-mediated post-translational modification of cysteine thiols of the proteins and peptides in which cysteine is converted to nitrosothiol [3,16]. SNO plays a major role in the localization and activity of a wide range of key enzymes and receptors in physiological and pathological conditions, leading to modulation of many signaling pathways, axonal transport, synaptic plasticity, and protein assembly [3,16,18,19,20]. Aberrant SNO signaling may contribute to the progression of many neurodegenerative [21,22,23,24], neurodevelopmental [3,16], and neuropsychiatric disorders [16,25].

In the present study, we mapped the S-nitroso-proteome in an ASD model based on InsG3680(+/+) mutation of the *Shank3* gene using SNOTRAP-based mass spectrometry (MS) technology followed by systems biology analysis combined with bioinformatics. Interestingly, our analysis of the SNO proteins revealed enrichment of several mitochondrial processes, such as the respiratory electron transport chain, the ATP metabolic process, regulation of the neuron apoptotic process, and the cellular response to oxidative stress. This prompted us to suggest that altered SNO signaling may lead to modulation of mitochondrial proteins that ultimately would result in mitochondrial dysfunction contributing to the pathogenesis of ASD.

## 2. Methods and Materials

### 2.1. Cortical Tissue Preparation for MS Analysis

Cortex tissues were isolated from Juvenile InsG3680(+/+) mutant mice following decapitation during the day time. Three to four cortex tissue samples from 3–4 mice were pooled for each biological replicate. Furthermore, tissues were homogenized on ice in a freshly prepared lysis buffer: 250 mM HEPES-NaOH, 1 mM EDTA, 20 mM IAM, 0.1 mM neocuproine, 1% NP-40, 1% protease-I cocktail, pH 7.6–7.7. The homogenates were centrifuged (12,000× *g* for 10 min at 4 °C), the supernatant was collected, and protein concentration was estimated by Bradford assay (Bio-Rad, Hercules, CA, USA, Cat. No. 500-0006). Next, samples were alkylated with 30 mM IAM in the dark at 37 °C in the presence of 2.5% SDS. After the alkylation step, samples were washed 2 times with 3 times the volume of 8 M urea and once with 50 mM HEPES (pH 7.6–7.7) by centrifugation at 5000× *g* for 30 min at 4 °C with 10,000 MWCO spin filters pre-rinsed once with water (Satorious AG, Gottingen, Germany, Cat. No. VS15T01). Then, SNOTRAP labelling stock solutions were added to all samples to reach a final concentration of 1.25 mM. Next, all samples were incubated for 1.5 h in SNOTRAP solution at 25 °C. Following the labelling, reagents were excluded by three consecutive washes with a 50 mM HEPES (pH 7.7) buffer with 10,000 filters. Following on to the ultrafiltration, each sample was incubated with 150–200 μL pre-rinsed streptavidin agarose beads (Pierce, Cat. No. 20349) for 1 h at room temperature. After washing the beads, proteins were eluted with 10 mM TCEP in 50 mM HEPES, pH 7.7 and then alkylated with 8-10 mM IAM. All samples were prepared at room temperature in the dark. 

### 2.2. MS Analysis

The MS proteomics data, which we generated previously, were taken from the ProteomeXchange Consortium database (http://proteomecentral.proteomexchange.org, accessed on 2018) via the PRIDE partner repository with the dataset identifier <PXD006907> for ASD.

### 2.3. Bioinformatics and Systems Biology Analysis

For the systems biology analysis of the biological processes (BP), molecular functions (MF), cellular components (CC), and the protein–protein interaction network of the SNO proteins, we used the search tool for the interacting proteins (STRING, version 11.0; http://string-db.org, accessed on 2020). The Benjamini–Hochberg correction was used to calculate the false discovery rate (FDR). Terms with FDR values below 0.05 were accepted. Medium reliability interactions (score > 0.4) from the neighborhood, gene fusion, co-occurrence, co-expression, experiments, databases, and text mining lists were used. Cytoscape V3.3.0 software was used for visualization of the protein–protein interaction. GraphPad PRISM 8 software was used to generate the schematic figures. GO analysis includes the BP, MF, and CC analyses.

## 3. Results

### 3.1. Systems Biology Analysis of the S-Nitroso-Proteome in the ASD Mouse Model

Mapping and identification of the SNO proteins in the brain of the *Shank3* mouse model of ASD utilizing SNOTRAP-based mass spectrometry technology revealed a total of 550 proteins that were modulated by SNO. This was followed by systems biology analysis of the SNO proteins to dissect the BP, MF, and CC that are enriched among the SNO proteins. The analysis revealed enrichment of several processes and functions related to the mitochondria.

**Figure 1:**
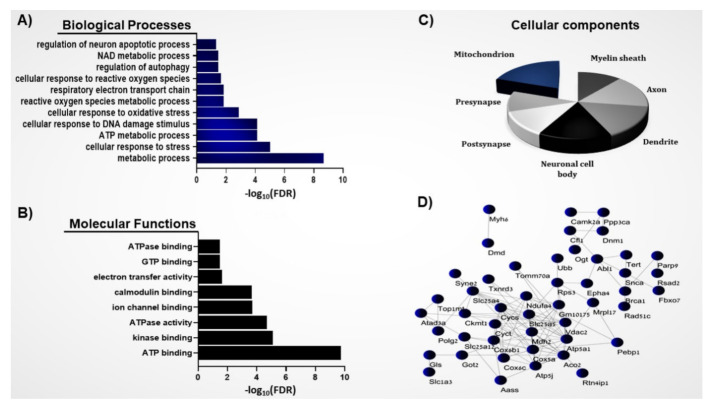
System biology analysis of the S-nitroso-proteome in the ASD mouse model. (**A**) BP, (**B**) MF, and (**C**) CC analyses were conducted on the SNO proteins. Bars represent the −log10 of the Benjamini-corrected false discovery rate (FDR). (**D**) Network analysis was conducted on the mitochondrial SNO proteins (*n* = 63).

BP analysis of the SNO proteins showed enrichment of several mitochondrial processes, such as regulation of the neuron apoptotic process (FDR = 0.0182), the ATP metabolic process (FDR = 7.35 × 10^−5^), the respiratory electron transport chain (FDR = 0.0149), the reactive oxygen species metabolic process (FDR = 0.0326), and the cellular response to oxidative stress (FDR = 0.0013) (Figure 1A). At the molecular level, the MF analysis of the SNO proteins demonstrated the enrichment of multiple molecular functions related to the mitochondria, such as electron transfer activity (FDR = 0.0223), ATPase activity (FDR = 1.90 × 10^−5^), ATP binding (FDR = 1.82 × 10^−10^), and ATPase binding (FDR = 0.0316) (Figure 1B). CC analysis of the SNO proteins revealed that the proteins were highly enriched in the mitochondria (FDR = 1.16 × 10^−5^). A total of 63 SNO proteins were found to be functionally localized in the mitochondria (Table 1). The proteins were also enriched in the dendrite (FDR = 2.43 × 10^−18^), axon (FDR = 1.97 × 10^−9^), presynapse (FDR = 2.23 × 10^−6^), postsynapse (FDR = 2.03 × 10^−7^), and others (Figure 1C).

### 3.2. Protein–Protein Interaction Network of the Mitochondrial SNO Proteins

We used the gene ontology (GO) classification system to classify the proteins into different clusters based on their enriched cellular components. This analysis revealed a distinct cluster of proteins (Figure 1D) that are involved in different mitochondria-associated processes, such as the respiratory electron transport chain, the ATP metabolic process, regulation of the neuron apoptotic process, and the cellular response to oxidative stress. The cluster included CAMK2A, COX6C, SNCA, GOT2, DNM1, TERT, UBB, RAD51C, OGT, SLC25A12, MYH6, ATP5J, and other proteins (Figure 1D).

## 4. Discussion

In the present study, the GO analysis of the SNO proteome in the ASD mouse model revealed SNO-related enrichment of mitochondria-associated processes, such as the respiratory electron transport chain, the ATP metabolic process, regulation of the neuron apoptotic process, the cellular response to oxidative stress, and the reactive oxygen species metabolic process. Over recent years, several lines of observation have implicated the mitochondria in the pathophysiology of ASD [6,9,10,26]. Thus, we suggest that aberrant SNO signaling induced by the *Shank3* mutation might provide a mechanistic explanation underlying the mitochondrial dysfunction in ASD.

NO is a signaling molecule, essential for the regulation of multiple neuronal and synaptic functions [3,16,19]. NO affects cellular signaling through the formation of cyclic GMP, protein S-nitrosylation (SNO), tyrosine nitration, and the formation of S-nitrosoglutathione (GSNO). SNO is a reversible NO-mediated post-translational modification in which cysteine is converted into nitrosothiol. SNO regulates the activity and localization of a wide range of key enzymes and receptors leading to modulation of cell signaling pathways, neuronal functions, and synaptic plasticity [16,22,27,28]. Thus, aberrant SNO is implicated in neuropathology, such as Alzheimer’s disease (AD) [16,21,22,23,24], Parkinson’s disease (PD) [16,29], schizophrenia [16,25]. Regarding ASD, we have recently discovered for the first time the NO-related molecular changes and SNO signaling in the brains of the mouse model of ASD based on the InsG3680(+/+) mutation of *Shank3* [3]. SNO is dynamically regulated by the amount of the NO produced from L-arginine by the enzymatic activity of the neuronal nitric oxide synthase (nNOS). In the InsG3680(+/+) mice, increased NO output was observed at the locus of the pathology resulting in changes of the SNO proteome that affected neuronal and synaptic processes known to be involved in ASD [3,16]. Interestingly, in this study, we discovered several mitochondrial processes and proteins that were modulated by SNO.

**Figure 2:**
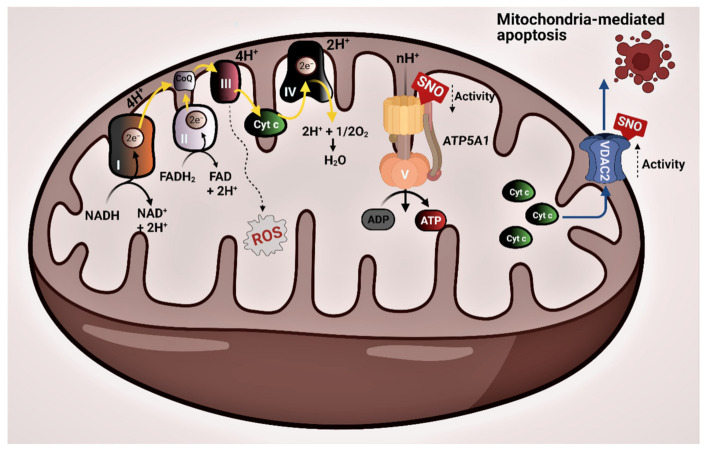
Schematic figure showing the respiratory electron transport chain (ETC) that drives the ATP synthesis. Electrons from NADH are transferred to oxygen by a series of unique and large protein complexes (I-IV) in the inner mitochondrial membrane, which creates the electrochemical gradient required for ATP synthesis by ATP5A1. ATP5A1: mitochondrial ATP synthase produces ATP from ADP in the presence of a proton gradient generated by the ETC. S-nitrosylation of ATP5A1 inhibits its activity. VDAC2: voltage-dependent anion-selective channel protein 2. S-nitrosylation of VDAC2 increases its activity. ROS: Reactive oxygen species, a byproduct of the ETC.

Enrichment of the ATP metabolic process and respiratory electron transport chain by the SNO proteins is demonstrated in Figure 1A. Mitochondrial energy production, which occurs through OXPHOS, requires the activity of five distinct enzyme complexes termed the electron transport chain (ETC), which are embedded in the inner membrane of the mitochondria. These multimeric complexes participate in the generation of the proton gradient in the mitochondrial intermembrane space, essential for the production of ATP: complex I (NADH dehydrogenase), complex II (succinate dehydrogenase), complex III (cytochrome *bc1* complex), complex IV (cytochrome *c* oxidase (COX)), and complex V (ATP synthase) [30] (Figure 2). Evaluation of the post-mortem brain samples of children with ASD showed alterations in the steady-state levels of complexes I-IV in the cerebellum, temporal cortex, frontal cortex, thalamus, and cingulate gyrus [6,26,30,31]. In the cerebellum, the investigators found decreased levels of complexes III and V. The expressions of complex I in the frontal cortex and of complexes II, III, and V in the temporal cortex of the developing brain of the autistic subjects were decreased compared to their neurotypical counterparts [26]. Another postmortem analysis assessed Brodmann area 21 within the lateral temporal lobe of the ASD brain [31]. Brodmann area is responsible for the processing of auditory, language, and social perception, and it has been involved in the manifestations of ASD [31]. Similar to the prior works, the researchers observed decreased protein levels of complexes I, III, IV, and V and impaired activities of complex I and IV in the autistic brain [31]. These observations were later confirmed in another postmortem study [30]. A reduced protein expression of several subunits of complex I, III, IV, and V in the motor cortex, cingulate gyrus, and thalamus of the autistic brain compared to the control was also reported [30]. Strikingly, the expression of ATP5A1 (complex V) of the ETC, which transforms adenosine diphosphate (ADP) into ATP, appeared to be reduced in all examined regions [6]. Our analysis showed that ATP5A1 undergoes SNO in the ASD mouse model (Figure 2). This could likely inhibit the activity of this complex, according to previous studies [32]. Based on these findings, we suggest that SNO might provide a mechanistic explanation for the observed mitochondrial alterations in ASD.

GO analysis of the SNO proteins revealed the enrichment of the reactive oxygen species metabolic process and the cellular response to oxidative stress. Oxygen metabolism by the ETC represents a major source of ROS [33] (Figure 2). Since these species contain an unpaired electron, they are highly reactive and unstable. Regulated levels of these free radicals are vital for key processes such as calcium homeostasis. However, they can potentially oxidize essential molecules, such as DNA, lipids, and proteins, which in turn would induce cell damage eventually leading to neuropathology [33]. In the brain, several antioxidant defense mechanisms are employed to counterbalance the levels of the free radicals and mitigate the damage they cause. These mechanisms include the synthesis of antioxidant enzymes, such as glutathione peroxidase and superoxide dismutase (SOD) [33,34,35]. Mitochondrial dysfunction, in particular, the deficits in the respiratory complexes of ETC, could lead to enhanced levels of ROS that cannot be sufficiently compensated by the antioxidant defense systems triggering oxidative stress [33]. Several reports implicated oxidative stress in the pathophysiology of ASD [36,37,38,39]. The cellular response to oxidative stress is a keystone in neuroinflammation that is considered one of the leading pathological factors of ASD [36]. Autistic children are found to be more vulnerable to oxidative stress due to the decreased levels of plasma and cellular glutathione [36]. Increased oxidative stress, decreased levels of SOD2, and DNA damage were observed in autistic brains in all the above-mentioned regions [11,26,30,31].

Regulation of the neuron apoptotic process was enriched among the SNO proteins. The mitochondria play a central role in the regulation of apoptosis [40]. Apoptosis is a vital mechanism that assesses the brain’s size and shape; it also regulates the proper wiring of the developing neuronal networks. However, aberrant activation of apoptotic pathways may impair neuroanatomy and brain maturation that may induce developmental disabilities and autism-like behaviors [41]. Magnetic resonance imaging (MRI) studies of total brain volume reported that autistic children have 5–10% atypical enlargement in some brain regions compared to neurotypical children [41]. Reduced volume of the corpus callosum and increased volume of the amygdala have also been observed in autistic children [42]. Abnormal structural organization in multiple brain regions was also reported in children and adolescents with ASD [43], all of which would indicate neuropathological activation of apoptotic pathways in ASD.

VDAC2 (voltage-dependent anion-selective channel protein 2) is one of the mitochondrial proteins that play an essential role in mitochondria-mediated apoptosis through its association with different proteins and ligands [44]. S-nitrosylation of this protein increases its activity [45]. Our data demonstrated that VDAC2 was S-nitrosylated in the ASD mouse model (Figure 2). Therefore, we suggest that SNO-mediated increased activity of VDAC2 may partially explain some of the mitochondrial dysfunctions leading to autistic symptoms. Taken together, NO can be regarded as a pathological factor implicated in the etiology of ASD, and this role of NO is at least partially associated with S-nitrosylation of the mitochondrial proteins leading to mitochondrial dysfunction.

In conclusion, altered SNO signaling induced by the *Shank3* mutation can target a wide range of proteins belonging to the mitochondria-related processes and functions, such as the respiratory electron transport chain, the cellular response to stress, and regulation of the neuron apoptotic process. Further study of the effects of S-nitrosylation of the proteins involved in these processes may help to unravel the neuropathological mechanism underlying the pathogenesis of ASD. The SNOTRAP-based MS approach combined with large-scale systems biology analysis facilitates the global profiling of the S-nitroso-proteome. This approach represents a powerful tool for the identification and characterization of the key proteins and processes related to ASD and might provide a therapeutic strategy for ASD in the future.

## 5. Conclusions

The SNOTRAP-based MS approach combined with large-scale systems biology analysis facilitates the global profiling of the S-nitroso-proteome. This approach represents a powerful tool for the identification and characterization of the key proteins and processes related to ASD. Thus, the present study revealed that altered SNO signaling induced by the *Shank3* mutation (which represents a well-established mouse model of ASD) can target a wide range of mitochondrial proteins. Several important mitochondria-related processes, including respiratory electron transport chain, cellular response to stress, regulation of neuron apoptotic process, and reactive oxygen species metabolic process, were SNO enriched. These data suggest that aberrant NO/SNO signaling is involved in the ASD pathology through alteration of the mitochondrial proteins and functions. Further study of the effects of S-nitrosylation on mitochondria may shed light on the molecular mechanisms underlying the pathogenesis of ASD and reveal new molecular targets for the treatment of this disorder.

## Figures and Tables

**Table 1:** The mitochondrial S-nitrosylated proteins (n = 63).

Accession ID	Protein’s Name
**Q03265**	ATP synthase subunit alpha, mitochondrial
**Q99KI0**	Aconitate hydratase, mitochondrial
**P05202**	Aspartate aminotransferase, mitochondrial
**P30275**	Creatine kinase U-type, mitochondrial
**P39053**	Dynamin-1
**P51881**	ADP/ATP translocase 2
**P63328**	Serine/threonine-protein phosphatase 2B catalytic subunit alpha isoform
**P18760**	Cofilin-1
**Q80VP2**	Spermatogenesis-associated protein 7 homolog
**Q8BMK0**	Centrosomal protein of 85 kDa
**P48754**	Breast cancer type 1 susceptibility protein homolog
**Q499E0**	Protein FAM5C
**D3Z7P3**	Glutaminase kidney isoform, mitochondrial
**O70337**	Bcl-2-interacting killer
**Q3U7U3**	F-box only protein 7
**Q924D0**	Reticulon-4-interacting protein 1, mitochondrial
**P62897**	Cytochrome c, somatic
**P51612**	DNA repair protein complementing XP-C cells homolog
**Q8CBB9**	Radical S-adenosyl methionine domain-containing protein 2
**P56564**	Excitatory amino acid transporter 1
**O70372**	Telomerase reverse transcriptase
**Q9CPQ1**	Cytochrome c oxidase subunit 6C
**P50171**	Estradiol 17-beta-dehydrogenase 8
**Q9R1M5**	NACHT, LRR and PYD domains-containing protein 5
**Q8BH59**	Calcium-binding mitochondrial carrier protein Aralar1
**P62908**	40S ribosomal protein S3
**Q02566**	Myosin-6
**Q03137**	Ephrin type-A receptor 4
**P0CG49**	Polyubiquitin-B
**O55042**	Alpha-synuclein
**P97450**	ATP synthase-coupling factor 6, mitochondrial
**P48962**	ADP/ATP translocase 1
**P08249**	Malate dehydrogenase, mitochondrial
**P28184**	Metallothionein-3
**P11531**	Dystrophin
**P70296**	Phosphatidylethanolamine-binding protein 1
**P00520**	Tyrosine-protein kinase ABL1
**P56391**	Cytochrome c oxidase subunit 6B1
**D3YXS5**	Kinesin-like protein KLP6
**P12787**	Cytochrome c oxidase subunit 5A, mitochondrial
**Q05A80**	Caprin-2
**Q149F5**	Transmembrane protein 71
**Q9D8P4**	39S ribosomal protein L17, mitochondrial
**P00015**	Cytochrome c, testis-specific
**Q9DAQ9**	Spermatogenesis-associated protein 19, mitochondrial
**Q8BX70**	Vacuolar protein sorting-associated protein 13C
**Q99MD6**	Thioredoxin reductase 3 (Fragment)
**Q60575**	Kinesin-like protein KIF1B
**Q6K1E7**	Gametogenetin-binding protein 1
**Q8R4U6**	DNA topoisomerase I, mitochondrial
**Q99K67**	Alpha-aminoadipic semialdehyde synthase, mitochondrial
**Q924H5**	DNA repair protein RAD51 homolog 3
**Q60930**	Voltage-dependent anion-selective channel protein 2
**P11798**	Calcium/calmodulin-dependent protein kinase type II subunit alpha
**Q8CAS9**	Poly [ADP-ribose] polymerase 9
**Q9QZM2**	DNA polymerase subunit gamma-2, mitochondrial
**Q8CGY8**	UDP-N-acetylglucosamine--peptide N-acetylglucosaminyltransferase 110 kDa subunit
**Q9CZW5**	Mitochondrial import receptor subunit TOM70
**Q60855**	Receptor-interacting serine/threonine-protein kinase 1
**Q62425**	NADH dehydrogenase [ubiquinone] 1 alpha subcomplex subunit 4
**Q80Y44**	Probable ATP-dependent RNA helicase DDX10
**Q925I1**	ATPase family AAA domain-containing protein 3
**Q6ZWQ0**	Nesprin-2

## Data Availability

The MS proteomics data, which we generated previously, were taken from the ProteomeXchange Consortium database (http://proteomecentral.proteomexchange.org, accessed on 2018) via the PRIDE partner repository with the dataset identifier <PXD006907> for ASD.

## References

[1] Shen L., Zhang K., Feng C., Chen Y., Li S., Iqbal J., Liao L., Zhao Y., Zhai J. (2018). iTRAQ-Based Proteomic Analysis Reveals Protein Profile in Plasma from Children with Autism. Proteom. Clin. Appl..

[2] Lord C., Elsabbagh M., Baird G., Veenstra-Vanderweele J. (2018). Autism spectrum disorder. Lancet.

[3] Amal H., Barak B., Bhat V., Gong G., Joughin B.A., Wang X., Wishnok J.S., Feng G., Tannenbaum S.R. (2018). Shank3 mutation in a mouse model of autism leads to changes in the S-nitroso-proteome and affects key proteins involved in vesicle release and synaptic function. Mol. Psychiatry.

[4] Monteiro P., Feng G. (2017). SHANK proteins: Roles at the synapse and in autism spectrum disorder. Nat. Rev. Neurosci..

[5] Zhou Y., Kaiser T., Monteiro P., Zhang X., Van der Goes M.S., Wang D., Barak B., Zeng M., Li C., Lu C. (2016). Mice with Shank3 Mutations Associated with ASD and Schizophrenia Display Both Shared and Distinct Defects. Neuron.

[6] Griffiths K.K., Levy R.J. (2017). Evidence of Mitochondrial Dysfunction in Autism: Biochemical Links, Genetic-Based Associations, and Non-Energy-Related Mechanisms. Oxid. Med. Cell. Longev..

[7] Hsiao E.Y. (2014). Gastrointestinal Issues in Autism Spectrum Disorder. Harv. Rev. Psychiatry.

[8] Rossignol D.A., Frye R.E. (2014). Evidence linking oxidative stress, mitochondrial dysfunction, and inflammation in the brain of individuals with autism. Front. Physiol..

[9] Frye R.E. (2020). Mitochondrial Dysfunction in Autism Spectrum Disorder: Unique Abnormalities and Targeted Treatments. Semin. Pediatr. Neurol..

[10] Frye R.E., Rossignol D.A. (2011). Mitochondrial dysfunction can connect the diverse medical symptoms associated with autism spectrum disorders. Pediatr. Res..

[11] Citrigno L., Muglia M., Qualtieri A., Spadafora P., Cavalcanti F., Pioggia G., Cerasa A. (2020). The Mitochondrial Dysfunction Hypothesis in Autism Spectrum Disorders: Current Status and Future Perspectives. Int. J. Mol. Sci..

[12] Osellame L.D., Blacker T.S., Duchen M.R. (2012). Cellular and molecular mechanisms of mitochondrial function. Best Pract. Res. Clin. Endocrinol. Metab..

[13] Zhao R.Z., Jiang S., Zhang L., Yu Z.B. (2019). Mitochondrial electron transport chain, ROS generation and uncoupling (Review). Int. J. Mol. Med..

[14] Valenti D., de Bari L., De Filippis B., Henrion-Caude A., Vacca R.A. (2014). Mitochondrial dysfunction as a central actor in intellectual disability-related diseases: An overview of Down syndrome, autism, Fragile X and Rett syndrome. Neurosci. Biobehav. Rev..

[15] Brorson J.R., Schumacker P.T., Zhang H. (1999). Nitric oxide acutely inhibits neuronal energy production. The Committees on Neurobiology and Cell Physiology. J. Neurosci..

[16] Tripathi M.K., Kartawy M., Amal H. (2020). The role of nitric oxide in brain disorders: Autism spectrum disorder and other psychiatric, neurological, and neurodegenerative disorders. Redox Biol..

[17] Bredt D.S., Snyder S.H. (1994). Nitric oxide: A physiologic messenger molecule. Annu. Rev. Biochem..

[18] Khaliulin I., Kartawy M., Amal H. (2020). Sex Differences in Biological Processes and Nitrergic Signaling in Mouse Brain. Biomedicines.

[19] Kartawy M., Khaliulin I., Amal H. (2020). Systems biology reveals reprogramming of the S-nitroso-proteome in the cortical and striatal regions of mice during aging process. Sci. Rep..

[20] Hamoudi W., von Lendenfeld F., Kartawy M., Mencer S., Suloh H., Khaliulin I., Amal H. (2021). Regional Differences in S-Nitrosylation in the Cortex, Striatum, and Hippocampus of Juvenile Male Mice. J. Mol. Neurosci..

[21] Amal H., Gong G., Gjoneska E., Lewis S.M., Wishnok J.S., Tsai L.-H., Tannenbaum S.R. (2019). S-nitrosylation of E3 ubiquitin-protein ligase RNF213 alters non-canonical Wnt/Ca+2 signaling in the P301S mouse model of tauopathy. Transl. Psychiatry.

[22] Nakamura T., Lipton S.A. (2016). Protein S-Nitrosylation as a Therapeutic Target for Neurodegenerative Diseases. Trends Pharm. Sci..

[23] Nakamura T., Prikhodko O.A., Pirie E., Nagar S., Akhtar M.W., Oh C.-K., McKercher S.R., Ambasudhan R., Okamoto S.-i., Lipton S.A. (2015). Aberrant protein S-nitrosylation contributes to the pathophysiology of neurodegenerative diseases. Neurobiol. Dis..

[24] Nakamura T., Tu S., Akhtar M.W., Sunico C.R., Okamoto S.-i., Lipton S.A. (2013). Aberrant protein S-nitrosylation in neurodegenerative diseases. Neuron.

[25] Nasyrova R.F., Ivashchenko D.V., Ivanov M.V., Neznanov N.G. (2015). Role of nitric oxide and related molecules in schizophrenia pathogenesis: Biochemical, genetic and clinical aspects. Front. Physiol..

[26] Chauhan A., Gu F., Essa M.M., Wegiel J., Kaur K., Brown W.T., Chauhan V. (2011). Brain region-specific deficit in mitochondrial electron transport chain complexes in children with autism. J. Neurochem..

[27] Foley T.D., Koval K.S., Olsen S.H., Gallagher A.G., Dennis E.R. (2017). Protein S-Nitrosylation: Possible Links between Psychophysiological Stress and Neurodegeneration. Free Radic. Biol. Med..

[28] Rizza S., Cardaci S., Montagna C., Di Giacomo G., De Zio D., Bordi M., Maiani E., Campello S., Borreca A., Puca A.A. (2018). S-nitrosylation drives cell senescence and aging in mammals by controlling mitochondrial dynamics and mitophagy. Proc. Natl. Acad. Sci. USA.

[29] Yao D., Gu Z., Nakamura T., Shi Z.-Q., Ma Y., Gaston B., Palmer L.A., Rockenstein E.M., Zhang Z., Masliah E. (2004). Nitrosative stress linked to sporadic Parkinson’s disease: S-nitrosylation of parkin regulates its E3 ubiquitin ligase activity. Proc. Natl. Acad. Sci. USA.

[30] Anitha A., Nakamura K., Thanseem I., Matsuzaki H., Miyachi T., Tsujii M., Iwata Y., Suzuki K., Sugiyama T., Mori N. (2013). Downregulation of the expression of mitochondrial electron transport complex genes in autism brains. Brain Pathol..

[31] Tang G., Rios P.G., Kuo S.-H., Akman H.O., Rosoklija G., Tanji K., Dwork A., Schon E.A., Dimauro S., Goldman J. (2013). Mitochondrial abnormalities in temporal lobe of autistic brain. Neurobiol. Dis..

[32] Chang A.H., Sancheti H., Garcia J., Kaplowitz N., Cadenas E., Han D. (2014). Respiratory substrates regulate S-nitrosylation of mitochondrial proteins through a thiol-dependent pathway. Chem. Res. Toxicol..

[33] Siddiqui M.F., Elwell C., Johnson M.H. (2016). Mitochondrial Dysfunction in Autism Spectrum Disorders. Autism Open Access.

[34] Dringen R., Hirrlinger J. (2003). Glutathione pathways in the brain. Biol. Chem..

[35] Lee K.H., Cha M., Lee B.H. (2020). Neuroprotective Effect of Antioxidants in the Brain. Int. J. Mol. Sci..

[36] Bjørklund G., Meguid N.A., El-Bana M.A., Tinkov A.A., Saad K., Dadar M., Hemimi M., Skalny A.V., Hosnedlová B., Kizek R. (2020). Oxidative Stress in Autism Spectrum Disorder. Mol. Neurobiol..

[37] Nadeem A., Ahmad S.F., Attia S.M., Al-Ayadhi L.Y., Al-Harbi N.O., Bakheet S.A. (2019). Dysregulated enzymatic antioxidant network in peripheral neutrophils and monocytes in children with autism. Prog. Neuro-Psychopharmacol. Biol. Psychiatry.

[38] Eshraghi R.S., Deth R.C., Mittal R., Aranke M., Kay S.-I.S., Moshiree B., Eshraghi A.A. (2018). Early disruption of the microbiome leading to decreased antioxidant capacity and epigenetic changes: Implications for the rise in autism. Front. Cell. Neurosci..

[39] Wang Y., Zhao S., Liu X., Zheng Y., Li L., Meng S. (2018). Oxytocin improves animal behaviors and ameliorates oxidative stress and inflammation in autistic mice. Biomed. Pharmacother..

[40] Mayer B., Oberbauer R. (2003). Mitochondrial regulation of apoptosis. News Physiol. Sci..

[41] Wei H., Alberts I., Li X. (2014). The apoptotic perspective of autism. Int. J. Dev. Neurosci..

[42] Amaral D.G., Schumann C.M., Nordahl C.W. (2008). Neuroanatomy of autism. Trends Neurosci..

[43] Geschwind D.H., Levitt P. (2007). Autism spectrum disorders: Developmental disconnection syndromes. Curr. Opin. Neurobiol..

[44] Shoshan-Barmatz V., De Pinto V., Zweckstetter M., Raviv Z., Keinan N., Arbel N. (2010). VDAC, a multi-functional mitochondrial protein regulating cell life and death. Mol. Asp. Med..

[45] Zahid S., Khan R., Oellerich M., Ahmed N., Asif A.R. (2014). Differential S-nitrosylation of proteins in Alzheimer’s disease. Neuroscience.

